# Exposure-response analyses for belantamab mafodotin in combination with bortezomib and dexamethasone in patients with relapsed/refractory multiple myeloma from DREAMM-6 Arm B and DREAMM-7

**DOI:** 10.1038/s41416-026-03437-7

**Published:** 2026-04-13

**Authors:** Theodoros Papathanasiou, Xi Chen, Fernando Carreno, Astrid McKeown, Sumita Roy-Ghanta, Lydia Eccersley, Ravi Kasinathan, Nashita Patel, Geraldine Ferron-Brady

**Affiliations:** 1 Clinical Pharmacology, Modelling & Simulation, GSK, Baar, Switzerland; 2 Clinical Pharmacology, Modelling & Simulation, GSK, Upper Providence, PA USA; 3 Oncology Clinical Development, GSK, Stevenage, UK; 4 Oncology Clinical Development, GSK, London, UK; 5 Oncology Clinical Development, GSK, Upper Providence, PA USA

**Keywords:** Myeloma, Targeted therapies

## Abstract

**Background:**

Belantamab mafodotin, bortezomib and dexamethasone (BVd) demonstrated clinical activity in the phase I/II DREAMM-6 (Arm B) study and significant clinical benefit in the phase III DREAMM-7 study for patients with relapsed/refractory multiple myeloma (RRMM) and ≥1 prior line of therapy.

**Methods:**

Population pharmacokinetic-derived Cycle 1 (C1) belantamab mafodotin exposures were used to evaluate exposure-efficacy/exposure-safety relationships across multiple doses and schedules for benefit-risk assessment.

**Results:**

Belantamab mafodotin C1 exposure was positively associated with response endpoints and grade ≥2/ ≥3 ophthalmic exam findings (OEFs), but not grade ≥2/ ≥3 ocular adverse events (oAEs) or best-corrected visual acuity (BCVA) worsening to 20/50 or worse in both eyes. Probability of very good partial response or better (≥VGPR) was higher than grade ≥3 oAEs/BCVA worsening in both eyes across C1 exposures; efficacy improved at higher C1 exposures without increased OEF risk. Model-based benefit-risk assessment showed a belantamab mafodotin starting dose of 1.9 mg/kg instead of 2.5 mg/kg would result in lower probability of ≥VGPR without reduction in BCVA worsening in both eyes/grade ≥3 oAEs.

**Conclusions:**

An initial belantamab mafodotin dose of 2.5 mg/kg for BVd yields deeper responses with minimal change in safety outcomes versus 1.9 mg/kg for patients with RRMM.

## Introduction

Belantamab mafodotin is an antibody-drug conjugate (ADC) that binds to B-cell maturation antigen (BCMA) [1], which is expressed by multiple myeloma (MM) tumor plasma cells [1, 2]. Belantamab mafodotin is composed of an afucosylated humanized anti-BCMA immunoglobulin G1 (IgG1) monoclonal antibody conjugated to the microtubule inhibitor monomethyl auristatin F via a protease-resistant maleimidocaproyl linker (cys-mcMMAF) [1], and is being evaluated in combination with standards of care in several ongoing clinical studies of MM. Belantamab mafodotin plus bortezomib and dexamethasone (BVd) is under investigation in Arm B of the phase I/II, open-label DREAMM-6 (NCT03544281) study evaluating the safety, tolerability, and clinical activity of BVd at multiple dosing regimens in patients following at least 1 prior line of therapy (LOT) [3]. Additionally, the multicenter, open-label, randomized phase III DREAMM-7 (NCT04246047) study is evaluating BVd in patients with relapsed/refractory MM (RRMM) in their second LOT or later [4]. Interim analyses of data from DREAMM-7 have shown that, compared with daratumumab, bortezomib and dexamethasone (DVd) therapy, treatment with BVd resulted in a significant benefit in progression-free survival (PFS) and overall survival (medians not reached in either arm; hazard ratio (HR) 0.58 [95% confidence interval (CI) 0.43–0.79], P = 0.00023) [5, 6]. Based on these findings, BVd has been approved in the United Kingdom, Japan, the EU, and Canada for the treatment of patients with RRMM who have received at least 1 prior LOT [7–10].

Despite these promising preliminary findings, it is important to note that ocular adverse events (AEs) have previously been reported in patients treated with belantamab mafodotin and appear to be a class effect of MMAF-containing ADCs [11, 12]. The Keratopathy and Visual Acuity (KVA) scale incorporates both ophthalmic exam findings and changes in best-corrected visual acuity (BCVA) into a single scale which has been used to manage these events through changes in dose and/or extension of the dosing interval. A previous exposure-response (E-R) analysis has indicated that both efficacy and safety, including ocular AEs, may be associated with Cycle 1 belantamab mafodotin exposure [13]. However, the analysis evaluated belantamab mafodotin monotherapy in patients with RRMM at later LOTs, and there remains a need to characterize these relationships in combination regimens in patients with RRMM who have received at least 1 prior LOT to properly inform dosing choices.

The aim of the E-R analyses was to confirm that the starting dose of belantamab mafodotin used in the BVd regimen of the DREAMM-7 study was appropriate for maximizing efficacy while keeping AEs at acceptable levels. More specifically, the E-R analyses aimed to delineate the relationship between belantamab mafodotin and/or cys-mcMMAF payload exposures and efficacy/safety outcomes across range of doses (1.9 to 3.4 mg/kg) and schedules in BVd-treated patients enrolled in DREAMM-6 (Arm B only) and DREAMM-7, while also identifying and evaluating other covariates of interest.

## Methods

### Study designs, treatment regimens, and patient populations

DREAMM-6 (Arm B), a phase I/II dose-escalation and expansion study, investigated multiple dosing regimens of BVd in patients with RRMM who had at least one prior LOT as described in the **Supplement**. The belantamab mafodotin treatment doses/schedules (all administered by intravenous infusion) were: 3.4 mg/kg every 21-day cycle, i.e., every 3 weeks (3.4 mg/kg Q3W); 1.7 mg/kg on Day 1 Q3W and 1.7 mg/kg on Day 8 Q3W (3.4 mg/kg split Q3W); 2.5 mg/kg every 21-day cycle (2.5 mg/kg Q3W); 2.5 mg/kg on alternate 21-day cycles, i.e., every 6 weeks, e.g., Cycle 1, 3, 5, 7 (2.5 mg/kg Q6W); 2.5 mg/kg in Cycle 1 then 1.9 mg/kg Q6W starting with Cycle 3 (2.5 mg/kg S/D to 1.9 Q6W); 1.25 mg/kg on Day 1 Q3W and 1.25 mg/kg on Day 8 Q3W (2.5 mg/kg split Q3W); 1.9 mg/kg every 21-day cycle (1.9 mg/kg Q3W) and 1.9 mg/kg on alternate 21-day cycles (1.9 mg/kg Q6W). After 8 cycles of combination therapy, belantamab mafodotin monotherapy was continued until disease progression, death, unacceptable toxicity, patient withdrawal, or end of study [3].

DREAMM-7, an ongoing phase III study, is investigating BVd versus DVd in patients with a confirmed diagnosis of MM who had previously been treated with at least one LOT and had documented disease progression [5]. In the BVd group, belantamab mafodotin is administered by intravenous infusion at a dose of 2.5 mg/kg on Day 1 every 21-day cycle. After 8 cycles of combination therapy, belantamab mafodotin monotherapy is continued until disease progression, death, unacceptable toxicity, patient withdrawal, or end of study [4, 5].

Additional inclusion and exclusion criteria for DREAMM-6 Arm B and DREAMM-7 are presented in the **Supplement**.

### E-R analyses

E-R analyses (i.e., statistical analysis of the relationship between response and pharmacokinetic [PK] measures of drug exposure) were performed using Cycle 1 exposure measures for belantamab mafodotin and cys-mcMMAF (safety E-R analyses only), which were derived from individual post hoc estimates obtained from the population PK analyses of DREAMM-6 Arm B (described in the **Supplement**) and DREAMM-7 [14]. Patients who received BVd in DREAMM-6 Arm B or DREAMM-7 were included in exposure-safety analyses if they received at least one dose of belantamab mafodotin and had a measurable PK sample available. Patients were included in the exposure-efficacy analyses population if, in addition to the above, they had measurable disease at baseline.

For belantamab mafodotin, exposure measures included: (i) maximum plasma concentration (C_max_) after the first dose; (ii) plasma concentration at the end of the first cycle (Day 21); and (iii) average plasma concentration (C_avg_) over the first cycle (21 days), with a focus on C_avg_ as the main determinant of exposure. For cys-mcMMAF (used in the safety E-R analyses only), C_max_ after the first dose and C_avg_ over the first cycle were derived.

### Endpoints for efficacy analyses

Efficacy E-R analyses were performed using the following endpoints: (i) PFS, defined as the time from date of randomization until the earliest date of confirmed disease progression (PD) or death due to any cause; (ii) duration of response (DOR), defined as the time from first documented evidence of a partial response (PR) or better until PD or death due to any cause; (iii) the probability of response, assessed by International Myeloma Working Group criteria including the probability of overall response, probability of a very good PR or better ( ≥VGPR), probability of a complete response or better ( ≥CR), and the probability of minimal residual disease (MRD) negativity for patients with ≥CR (DREAMM-7 only; unavailable for DREAMM-6 Arm B); and (iv) time to response (TTR), defined as the time between the date of randomization and the first documented evidence of response (PR or better; ≥PR) among patients who achieved a confirmed ≥PR.

### Endpoints considered for safety analyses

Ocular endpoints considered for safety E-R analyses are referred to as ocular AEs if they were assessed per the Common Terminology Criteria for Adverse Events (CTCAE), and as ophthalmic exam findings (OEFs; i.e, corneal exam findings and BCVA changes) if they were assessed per the KVA scale. These included: (i) the probability of any grade ≥2 or grade ≥3 ocular AEs by CTCAE (DREAMM-7 only, due to differing reporting criteria [keratopathy included in reporting for DREAMM-6 Arm B]); (ii) the time to first grade ≥2 or grade ≥3 ocular AE (CTCAE version 4.03; DREAMM-7 only), (iii) the probability of any grade ≥2 or grade ≥3 OEFs, assessed using the KVA scale (overall, corneal exam findings, BCVA change); (iv) the time to first grade ≥2 or grade ≥3 OEFs (KVA scale); (v) the probability of worsening BCVA, defined as a ≥ 0.3-point change in the logarithm of the minimum angle of resolution (logMar) for the better-seeing eye; and (vi) worsening of BCVA on one eye or both eyes to 20/50 or worse regardless of BCVA at baseline (DREAMM-7 only; data unavailable for DREAMM-6 Arm B).

Additional safety endpoints that were considered for the E-R analysis (dependent on their incidence) included the probability of: (i) infusion-related reactions (IRRs; a hybrid term for various AEs of special interest [AESI] that occurred within 24 hours of infusion and led to dose modifications or discontinuation); (ii) grade ≥3 thrombocytopenia (assessed using clinical laboratory data); (iii) grade 3–4 treatment-emergent AEs (TEAEs); (iv) fatal serious AEs (SAEs); and (v) dose modifications due to TEAEs (either dose discontinuations, dose reductions, or dose delays/interruptions).

Efficacy and safety endpoints with an incidence of ≤10% or ≥90% (where applicable) were not included in the E-R analyses.

### Statistical analyses

Time-to-event endpoints were visualized using Kaplan–Meier methodology stratified by exposure quartiles, and their relationship to belantamab mafodotin/cys-mcMMAF exposure was characterized using Cox proportional hazards modeling. The relationship between exposure and other endpoints (probability of occurrence, e.g., ocular AE) was characterized using logistic regression modeling.

For these models, covariates of interest included patient demographics (including geographical region), clinical characteristics, disease-related factors, presence of ocular conditions at baseline (i.e., dry eye, keratopathy, BCVA in better and worse eyes), thrombocytopenia, and planned dosing schedule (Supplementary Table 1). Covariate analyses were performed using stepwise forward addition and backward elimination for all modeling analyses (Cox proportional and logistic regression). During this process, individual covariates were first included in the model (univariate analysis) and those that showed significance at an α of 0.01 (corresponding to a reduction in the objective function value [OFV] of ≥6.64 for 1 degree of freedom) were included in the subsequent stepwise addition. This iterative process involved adding the most significant covariate into the model first, using this model to identify the next (most significant) candidate out of the remaining covariates, adding this into the model, and continuing this process until all significant covariates had been identified and included. However, for exposure covariates only the most significant (at the step where it is added to the model) was included.

After the full model was defined, the significance of each covariate was tested by removal (one at a time) from the full model (backward elimination). A covariate was retained in the model if it was significant at the 0.001 level (corresponding to an increase in the OFV of >10.83 points, upon removal, for 1 degree of freedom). The elimination steps were repeated until all non-significant covariates were excluded, and the final model defined. However, non-significant covariates could be retained in the final model if there was a strong pharmacological or physiological rationale for inclusion. In addition, if an endpoint included a belantamab mafodotin Cycle 1 exposure measure other than ADC C_avg_ in the final model, an alternative model replacing the exposure measure with ADC C_avg_ was also generated to allow model comparison across endpoints. If the alternative model using ADC C_avg_ provided a similar fit to the data as the model using a different exposure parameter, ADC C_avg_ superseded the other parameter.

All E-R analyses and post-processing of results were performed using R (version 4.1.3 or higher). Integrated E-R analysis plots were generated for visual clinical benefit-risk assessment using efficacy data from DREAMM-7. The integrated analyses focused on the confirmatory phase III trial data from DREAMM-7 due to the availability of data relating to relevant AEs (BCVA worsening to 20/50 or worse, grade ≥3 ocular AE per CTCAE and grade ≥3 OEFs). For calculation of odds ratios (ORs) or HRs for endpoints in the E-R analysis, a typical patient was defined as a 65-year-old male weighing 75 kg (body mass index 27 kg/m^2^), with mild renal impairment (estimated glomerular filtration rate of 70 mL/min), and normal liver function (alanine and aspartate aminotransferase levels of 20 IU/L, total bilirubin 0.4 mg/dL), with median baseline levels of soluble BCMA, IgG, and albumin of 50 ng/mL, 15 g/L, and 40 g/L, respectively. Changes from the typical exposure and covariate values that were used to compute ORs and HRs are described in the **Supplementary Methods**. For two-level categorical covariates, the OR corresponding to the flag being true (i.e., delta=1) is reported. For multi-level categorical covariates, the OR corresponding to the specified level compared with the most usual level in the dataset is reported.

Additional details on statistical modeling, stepwise covariate selection, representative R implementation, and computational environment are available in the **Supplementary Methods**.

## Results

### Patient population

Overall, 349 patients treated with BVd were included in the E-R analyses: 107 from DREAMM-6 (Arm B: 3.4 mg/kg Q3W n = 16; 3.4 mg/kg split Q3W n = 12; 2.5 mg/kg Q3W n = 18; 2.5 mg/kg Q6W n = 12; 2.5 mg/kg S/D to 1.9 Q6W n = 12; 2.5 mg/kg split Q3W n = 13; 1.9 mg/kg Q3W n = 12, and 1.9 mg/kg Q6W n = 12) and 242 from DREAMM-7. Patient demographics and disease characteristics are presented in Table 1. In brief, the majority of patients were ≥65 years of age (53.0%), male (56.4%), White (85.7%), had stage II or III disease (55.0%) and had a ‘standard’ cytogenetic risk profile (73.9%). A total of 159 patients (45.6%) resided in Europe.

**Table 1:** Baseline demographics and covariates

	Overall (N = 349)	DREAMM-6 Arm B[3, 26, 27] (n = 107)	DREAMM-7 (n = 242)
**Age group (years), n (%)**			
<65	164 (47.0)	43 (40.2)	121 (50.0)
65– <75	129 (37.0)	45 (42.1)	84 (34.7)
≥75	56 (16.0)	19 (17.8)	37 (15.3)
**Age (years)**			
Mean (SD)	64.5 (9.4)	64.8 (9.1)	64.4 (9.5)
Median (range)	65.0 (32.0–86.0)	66.0 (32.0–83.0)	64.5 (34.0–86.0)
**Baseline body weight (kg)**			
Mean (SD)	77.9 (17.8)	81.4 (19.9)	76.3 (16.6)
Median (range)	75.7 (43.7–170)	79.1 (46.5–170)	73.6 (43.7–133)
**Baseline body mass index (kg/m²)**			
Median (range)	27.1 (18.6–47.5)	26.8 (19.3–47.0)	27.1 (18.6–47.5)
**Sex, n (%)**			
Male	197 (56.4)	69 (64.5)	128 (52.9)
Female	152 (43.6)	38 (35.5)	114 (47.1)
**Race, n (%)**			
White	299 (85.7)	93 (86.9)	206 (85.1)
Black or African American	17 (4.9)	9 (8.4)	8 (3.3)
Asian	32 (9.2)	4 (3.7)	28 (11.6)
Native Hawaiian or other Pacific islander	1 (0.3)	1 (0.9)	0
**Region, n (%)**			
Europe	159 (45.6)	33 (30.8)	126 (52.1)
North America	32 (9.2)	22 (20.6)	10 (4.1)
North East Asia	26 (7.4)	0	26 (10.7)
Rest of the world	132 (38.0)	52 (48.6)	80 (33.1)
**MyelomaIg, n (%)**			
IgA	78 (22.3)	23 (21.5)	55 (22.7)
IgG	266 (76.2)	82 (76.6)	184 (76.0)
IgM	2 (0.6)	1 (0.9)	1 (0.4)
Other	3 (0.9)	1 (0.9)	2 (0.8)
**Revised International Staging System at screening, n (%)**			
I	155 (44.4)	53 (49.5)	102 (42.1)
II	167 (47.9)	37 (34.6)	130 (53.7)
III	25 (7.2)	17 (15.9)	8 (3.3)
Unknown	2 (0.6)	0	2 (0.8)
**Baseline ECOG, n (%)**			
0	174 (49.9)	53 (49.5)	121 (50.0)
1	157 (45.0)	46 (43.0)	111 (45.9)
2	18 (5.2)	8 (7.5)	10 (4.1)
**Cytogenetic risk categories, n (%)**^a^			
Standard	258 (73.9)	83 (77.6)	175 (72.3)
High Risk	90 (25.8)	24 (22.4)	66 (27.3)
**Extramedullary disease, n (%)**			
Yes	35 (10.0)	22 (20.6)	13 (5.4)
**Prior lines of therapy, n (%)**			
1	147 (42.1)	22 (20.6)	125 (51.7)
2	65 (18.6)	12 (11.2)	53 (21.9)
3	52 (14.9)	18 (16.8)	34 (14.0)
≥4	85 (24.4)	55 (51.4)	30 (12.4)
**Prior anti-CD38 treatment, n (%)**			
Yes	64 (18.3)	61 (57.0)	3 (1.2)
**Prior bortezomib treatment, n (%)**			
Yes	306 (87.7)	96 (89.7)	210 (86.8)
**Prior lenalidomide, n (%)**^**b**^			
Yes	127 (36.4)	NA	127 (52.5)
**Refractory to lenalidomide, n (%)**			
Yes	79 (22.6)	NA	79 (32.6)
**Refractory to both immunomodulatory drugs and proteasome inhibitors, n (%)**			
Yes	15 (4.3)	0	15 (6.2)
**Renal function, n (%)**			
Normal	126 (36.1)	53 (49.5)	73 (30.2)
Mild	144 (41.3)	32 (29.9)	112 (46.3)
Moderate	78 (22.3)	21 (19.6)	57 (23.6)
Severe	1 (0.3)	1 (0.9)	0
**Hepatic function, n (%)**			
Normal	305 (87.4)	86 (80.4)	219 (90.5)
Mild	41 (11.7)	19 (17.8)	22 (9.1)
Missing	3 (0.9)	2 (1.9)	1 (0.4)
**Dosing schedule, n (%)**^**c**^			
Reference standard dosing	288 (82.5)	46 (43.0)	242 (100)
Split dosing	25 (7.2)	25 (23.4)	0
Stretch dosing	24 (6.9)	24 (22.4)	0
Stepdown stretch dosing	12 (3.4)	12 (11.2)	0
**History of dry eye, n (%)**			
Yes	46 (13.2)	15 (14.0)	31 (12.8)
**Presence of keratopathy at baseline, n (%)**			
None	275 (78.8)	82 (76.6)	193 (79.8)
Mild	71 (20.3)	23 (21.5)	48 (19.8)
Moderate/Severe	3 (0.9)	2 (1.9)	1 (0.4)
**Baseline sBCMA (ng/mL)**			
Mean (SD)	89.5 (130)	89.8 (108)	89.4 (139)
Median (range)	42.7 (2.2–820)	44.7 (4.6–584)	39.1 (2.2–820)
**Baseline IgG (g/L)**			
Mean (SD)	19.5 (16.5)	17.3 (16.9)	20.5 (16.3)
Median (range)	15.6 (1.1–81.5)	12.2 (1.1–78.9)	18.2 (1.1–81.5)
**Baseline albumin (g/L)**			
Mean (SD)	39.7 (5.36)	37.3 (5.4)	40.8 (5.0)
Median (range)	40.0 (21.0–51.0)	38.0 (21.0–48.0)	41.0 (25.0–51.0)
**Baseline LDH (U/L)**			
Mean (SD)	237 (230)	277 (283)	219 (200)
Median (range)	191 (59–3020)	203 (118–2060)	186 (59–3020)
**Baseline beta-2-microglobulin (nmol/L)**			
Mean (SD)	306 (191)	335 (214)	293 (179)
Median (range)	261 (95–1530)	280 (136–1530)	253 (95–1530)
**Baseline M-protein (g/L)**			
Mean (SD)	19.5 (13.4)	19.6 (16.1)	19.5 (12.0)
Median (range)	17.0 (0.1–85.0)	16.5 (0.1–85.0)	17.9 (0.7–70.5)
**Baseline platelet count (x10**^**9**^**/L)**			
Mean (SD)	183 (69.9)	178 (66.3)	186 (71.5)
Median (range)	175 (45.0–620)	174 (45.0–407)	179 (62.0–620)
**Baseline BCVA best eye (logMAR)**			
Mean (SD)	0.038 (0.164)	0.008 (0.130)	0.051 (0.175)
Median (range)	0 (-0.20–2.00)	0 (-0.12–0.48)	0 (-0.20–2.00)
**Baseline BCVA worst eye (logMAR)**			
Mean (SD)	0.132 (0.331)	0.155 (0.473)	0.123 (0.244)
Median (range)	0 (-0.20–3.00)	0 (-0.12–3.00)	0 (-0.20–2.00)

*BCVA* best-corrected visual acuity, *CTCAE* Common Terminology Criteria for Adverse Events, *ECOG* Eastern Cooperative Oncology Group, *E-R* exposure-response, *Ig* immunoglobulin, *LDH* lactate dehydrogenase, *logMAR* logarithm of the minimum angle of resolution, *Q3W* every 3 weeks, *Q6W* every 6 weeks, *sBCMA* soluble B-cell maturation antigen, *SD* standard deviation, *S/D* step-down.

^a^‘Standard’ is defined by negative results for all high-risk abnormalities, and ‘high risk’ is defined by presence of at least one of the following; t(4;14), t(14;16), and del(17p13); n = 1 (0.3%) patient had ‘missing/not evaluable’ data.

^b^For DREAMM-6 Arm B study, n = 86 patients were reported as lenalidomide-exposed. However, at the time of the E-R analyses, all data for prior lenalidomide exposure in the DREAMM-6 Arm B study were treated as ‘missing’ or ‘not evaluable’.

^c^The E-R analyses tested dosing schedules in individual categories regardless of dose level. Reference standard: Q3W. Split: dosing split 50:50 between Days 1 and 8 Q3W. Stretch: Q6W. Stepdown stretch: Q6W with planned dose reduction at Cycle 3.

Patient characteristics were broadly balanced between the DREAMM-6 Arm B and DREAMM-7 populations, including baseline IgG, albumin, soluble BCMA (with means of 17.3 vs 20.5 g/L, 37.3 vs 40.8 g/L, and 89.8 vs 89.4 ng/mL, respectively), and baseline Eastern Cooperative Oncology Group score (0/1: 92.5% vs 95.9%), while baseline lactate dehydrogenase was slightly higher in DREAMM-6 Arm B (mean 277 vs 219 U/L). Notable differences in DREAMM-6 Arm B and DREAMM-7 populations included the presence of extramedullary disease (20.6% vs 5.4%, respectively), disease severity (Revised International Staging System Stage II: 34.6% vs 53.7%; Stage III: 15.9% vs 3.3%), number of prior lines of therapy (one: 20.6% vs 51.7%; at least four: 51.4% vs 12.4%), and prior anti-CD38 therapy (57.0% vs 1.2%).

### Efficacy E-R analyses

**Fig. 1:**
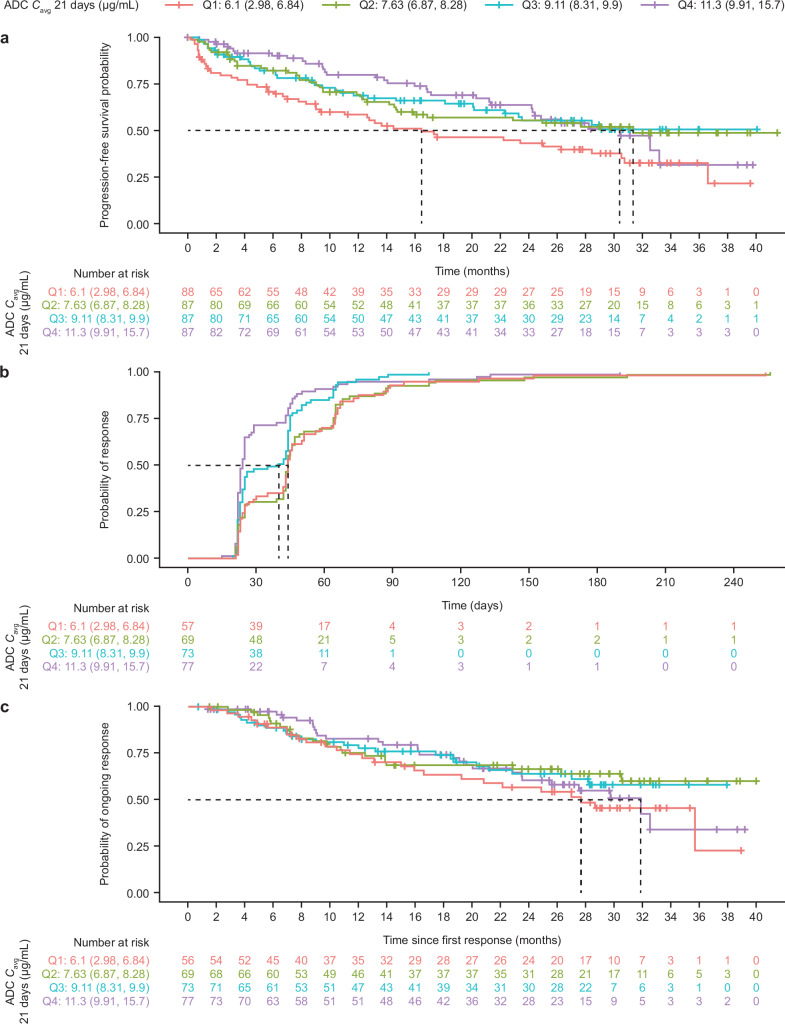
Kaplan–Meier plots by belantamab mafodotin C_avg_ quartiles (combined trials). PFS (**a**), TTR (**b**), DOR (**c**). ADC antibody-drug conjugate, C_avg_ average concentration, DOR duration of response, PFS progression-free survival, TTR time to response, Q quartile.

In analyses combining DREAMM-6 Arm B with DREAMM-7, the Kaplan–Meier curve for quartiles of Cycle 1 belantamab mafodotin C_avg_ showed that the shortest PFS was observed in the lowest exposure quartile (median C_avg_ quartiles ranged from 6.1 to 11.3 μg/mL), but no strong E-R trend in the curves was observed overall (Fig. 1A). Stepwise Cox regression modeling found exposure (C_avg_) to be significant in the univariate analyses (Supplementary Table 2); however, no significant association was retained between exposure measures and PFS when accounting for the effect of baseline covariates (prior anti-CD38 treatment, presence of extramedullary disease, and lactate dehydrogenase).

Stepwise Cox regression modeling indicated that higher belantamab mafodotin Cycle 1 C_avg_ was associated with a higher probability of overall response (OR 1.97 [95% CI 1.48, 2.69]), a higher probability of ≥VGPR (OR 1.95 [95% CI 1.54, 2.51]), and a shorter TTR (OR 1.33 [95% CI 1.19, 1.49]; Supplementary Tables 2 and 3 with univariate analysis results and final model information), though the Kaplan–Meier curve for TTR showed responses occurred within the first 2 cycles for all exposure quartiles (Fig. 1B). No clear E-R trend was observed for DOR other than a visually shorter but not statistically significant median DOR in the lowest C_avg_ quartile (Fig. 1C). Similar overall findings were observed in E-R analyses using only data from DREAMM-7 (Supplementary Table 4 and Supplementary Fig. 1).

Final models in the combined DREAMM-6 Arm B and DREAMM-7 analysis included prior anti-CD38 treatment as a covariate associated with shorter PFS and DOR, and a lower probability of responses (≥VGPR, ≥CR); extramedullary disease was associated with shorter PFS and a lower probability of responses (OR, ≥VGPR); higher baseline IgG was associated with a lower probability of ≥CR; and higher baseline lactate dehydrogenase was associated with shorter PFS. Baseline sBCMA levels, IgG type myeloma, high-risk cytogenetics, race (White, Asian, North East Asian, or Black/African American), geographical region, and age/age category (<65, 65 to <75, or ≥75 years) were not significantly associated with any efficacy endpoint. No covariates were associated with the probability of achieving MRD negativity at ≥CR response (tested in DREAMM-7 only), and dosing schedule (tested in the combined DREAMM-6 Arm B and DREAMM-7 population) was not associated with any of the efficacy endpoints analyzed. With the most notable exception being prior anti-CD38 treatment, which was only used by 1% of patients in the DREAMM-7 study and thus not evaluated as a covariate, the final model covariates and parameter estimates for DREAMM-7 exposure-efficacy analyses were generally consistent with the combined DREAMM-6 Arm B and DREAMM-7 analyses (Supplementary Table 5).

### Safety E-R analyses

The incidence of each safety endpoint is presented in Supplementary Table 6. Safety E-R analyses for ocular AE (CTCAE grade), OEFs graded by the KVA scale, additional AE and dose modification (any drug) endpoints are presented in Supplementary Table 7 (univariate analysis and final model information). E-R models for the probability of IRRs or fatal events were not performed due to the low incidence rate (≤10%) and a model for the probability of dose modification was not performed due to the high incidence rate (≥90%).

For exposure parameters in the combined DREAMM-6 Arm B and DREAMM-7 analyses, a higher belantamab mafodotin C_avg_ was associated with a higher probability of, and shorter time to grade ≥2 and grade ≥3 OEFs (KVA scale), grade ≥2 and grade ≥3 corneal exam findings, and grade ≥2 and grade ≥3 (higher probability only) BCVA events (KVA scale), a higher probability of worsening BCVA (as defined by delta logMar ≥0.3 in the better eye), as well as a higher probability of dose delays/interruptions. In analyses of DREAMM-7 only, Cycle 1 exposure was not associated with probability of or time to grade ≥2 or ≥3 ocular AE (CTCAE grade) or probability of BCVA worsening to 20/50 or worse in both eyes; Cycle 1 cys-mcMMAF C_max_ was associated with a higher probability of BCVA worsening in one eye (to 20/50 or worse).

Covariates included in the final models for endpoints assessed in the combined DREAMM-6 Arm B and DREAMM-7 analyses included baseline platelet count, which was inversely associated with the probability of grade ≥3 thrombocytopenia, and residing in Europe which was associated with a longer time to a grade ≥3 corneal exam finding, although this should be interpreted with caution due to the small sample sizes in other regions. Compared with standard dosing schedules (DREAMM-6 Arm B and DREAMM-7 combined), alternate dosing schedules (i.e., Q3W split, Q6W, and S/D Q6W; DREAMM-6 Arm B only) were associated with shorter time to occurrence of grade ≥2 corneal exam findings, but a longer time to grade ≥3 BCVA events (both KVA scale). Dosing schedule was not significantly associated with any of the other safety endpoints analyzed.

Covariates included in the final models for endpoints assessed in DREAMM-7 only were lower baseline BCVA (worst eye) which was associated with an increased probability of BCVA worsening in one eye (to 20/50 or worse) and lower baseline BCVA (better eye) which was associated with an increased probability of BCVA worsening in both eyes (to 20/50 or worse). Race, geographical region (except the association between residing in Europe and time to first grade ≥3 corneal exam finding), and age/age category were not significantly associated with any safety endpoint in the combined analysis or the DREAMM-7 only analysis.

### Integrated E-R analyses

**Fig. 2:**
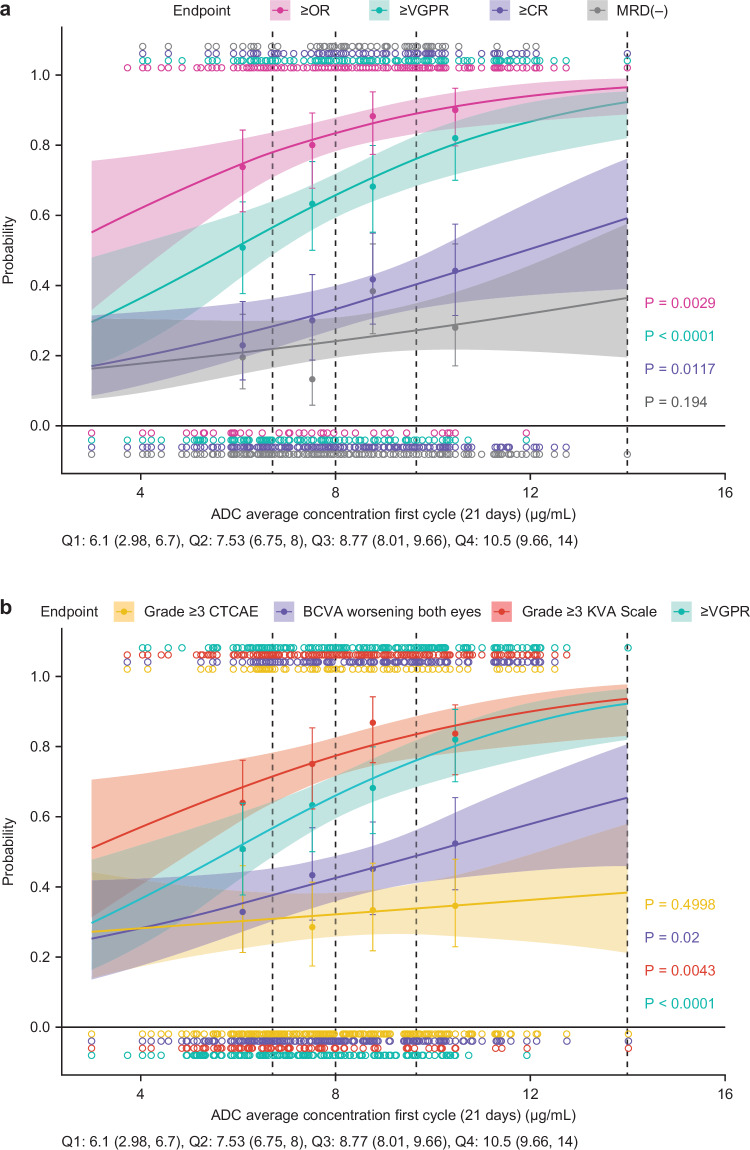
Integrated E-R analyses (DREAMM-7). Belantamab mafodotin *C*_avg_ relationship with probability of clinical response (**a**) and ocular safety overlaid with probability of ≥VGPR (**b**). ADC antibody-drug conjugate, AE adverse event, BCVA best-corrected visual acuity, *C*_avg_ average concentration, ≥CR complete response or better, CTCAE Common Terminology Criteria for Adverse Events, KVA keratopathy and visual acuity, MRD minimal residual disease, ≥OR objective response or better, Q quartile, ≥VGPR very good partial response or better.

Integrated E-R analyses between belantamab mafodotin C_avg_, and clinical responses ( ≥overall response, ≥VGPR, ≥CR and MRD negativity) and most clinically relevant safety endpoints on ocular events (grade ≥3 ocular AE, BCVA worsening in both eyes, grade ≥3 OEFs) are presented in Fig. 2A and B. Figure 2A presents a graphical overview of the probabilities of the various clinical responses together with Cycle 1 belantamab mafodotin C_avg_. While this figure does not take into consideration the potential confounding between disease factors that could impact response as well as exposure, the E-R analyses did. The analyses suggested positive E-R relationship with probability of objective response (for the combined DREAMM-7 with DREAMM-6 Arm B analysis only) and ≥VGPR. While the probability of objective response approached a plateau with the three upper quartiles of belantamab mafodotin exposure, the probability of ≥VGPR was greater with higher belantamab mafodotin exposure. No statistically significant association was found between Cycle 1 belantamab mafodotin exposure and probability of ≥CR or MRD negativity, though there was a trend for modest increases in the probability of these endpoints with higher exposures. The E-R curve of efficacy on probability of ≥VGPR was overlaid with E-R curves of safety on probability of grade ≥3 ocular AEs (CTCAE), probability of BCVA worsening to 20/50 or worse in both eyes, and probability of grade ≥3 OEF (KVA Scale) (Fig. 2B). Within the range of Cycle 1 belantamab mafodotin C_avg_, the probability of response ( ≥VGPR) was higher than the probability of grade ≥3 ocular AE (CTCAE) and the probability of BCVA worsening to 20/50 or worse in both eyes. There was an apparent positive relationship between Cycle 1 belantamab mafodotin C_avg_ and the probability of grade ≥3 OEF (KVA Scale).

Key model-predicted efficacy and safety endpoints for 1.9 mg/kg and 2.5 mg/kg belantamab mafodotin doses are summarized in Table 2. Overall, decreasing the initial dose of belantamab mafodotin from 2.5 mg/kg to 1.9 mg/kg resulted in a decrease in ≥VGPR of 14.9% and a decrease in the probability of grade ≥3 OEFs of 14.3%, with no change in the probability of BCVA worsening in both eyes or grade ≥3 ocular AE.

**Table 2:** Key model-predicted benefit-risk assessment findings.

Probability (%)	Belantamab mafodotin dose	
	1.9 mg/kg	2.5 mg/kg
≥VGPR^a^	53.1	68.0
≥CR^b^	34.7	34.7
Grade ≥3 ocular AEs (CTCAE)^b^	32.2	32.2
BCVA worsening in both eyes to 20/50 or worse^b^	37.9	37.9
Grade ≥3 OEFs (KVA)^a^	61.5	75.8

*AEs* adverse events, BCVA best-corrected visual acuity, *C*_*avg*_ average concentration, ≥CR complete response or better, *CTCAE* Common Terminology Criteria for Adverse Events, *KVA* keratopathy and visual acuity, *OEF* ophthalmic exam finding, *PK* pharmacokinetics, *≥VGPR* very good partial response or better.

Cycle 1 belantamab mafodotin C_avg_ for the 2.5 mg/kg dose (C_avg_: 7.8318 μg/mL) was derived from post hoc simulation using the final population PK model. Cycle 1 belantamab mafodotin C_avg_ for the 1.9 mg/kg dose (C_avg_: 5.9522 μg/mL) was calculated using a linear proportion assumption. Patients were assumed to have no extramedullary disease, no prior anti-CD38 treatment, and BCVA for the better seeing eye equal to zero. ≥CR and grade ≥3 ocular AEs (CTCAE) used the observed DREAMM-7 incidence rate since the final model is a null model.

^a^Combined DREAMM-6 Arm B and DREAMM-7 analyses.

^b^DREAMM-7 analyses: ≥CR and grade ≥3 ocular AEs used DREAMM-7 incidence rates since the final model was a null model.

## Discussion

This study reports the relationship between belantamab mafodotin Cycle 1 PK measures of drug exposure (doses ranging from 1.9 to 3.4 mg/kg) and efficacy/safety endpoints in patients with RRMM who received at least one prior line of anti-myeloma therapy and were treated with BVd in the DREAMM-6 Arm B and DREAMM-7 studies. The results of this study provide more comprehensive E-R analyses of belantamab mafodotin in RRMM than previously reported in patients in later LOTs, and represent the first pooled E-R analyses of belantamab mafodotin in combination with bortezomib and dexamethasone [13]. Findings from these integrated E-R analyses support that belantamab mafodotin at a 2.5 mg/kg starting dose drives deeper and faster clinical responses, without meaningful increases in the risk of key safety events (ocular AEs graded by CTCAE, BCVA changes to 20/50 or worse in one or both eyes, and grade ≥3 thrombocytopenia). While higher belantamab mafodotin exposure was associated with increased probability and earlier onset of grade ≥3 OEFs graded by the KVA scale, ocular AEs graded by CTCAE and BCVA changes to 20/50 or worse in both eyes are considered more clinically meaningful and relevant to patients. In general, ocular events were manageable with dose modifications (dose delays or reductions), enabling patients to continue treatment [5]. Multivariate models predicted that decreasing the initial dose of belantamab mafodotin from 2.5 mg/kg to 1.9 mg/kg would result in decreased probability of ≥VGPR, and no decrease in ≥CR; the probability of grade ≥3 OEFs (KVA scale) decreased with the lower dose, but probabilities of ocular AEs (CTCAE grade) and BCVA changes to 20/50 or worse in both eyes did not decrease. This indicates that in patients with RRMM the initial belantamab mafodotin dose of 2.5 mg/kg provides relatively higher benefit, with little to no additional safety risk, compared with an initial dose of 1.9 mg/kg.

Several covariates were associated with Cycle 1 belantamab mafodotin efficacy endpoints, including prior anti-CD38 treatment (reduced PFS, reduced DOR, and lower probability of ≥VGPR and ≥CR), extramedullary disease (reduced PFS and lower probability of overall response and ≥VGPR), and higher baseline IgG (lower probability of ≥CR). As higher baseline IgG and extramedullary disease are known prognostic factors indicative of higher MM disease burden [14, 15], lower efficacy may be expected in patients with these characteristics; however, disease burden-related factors (including IgG) have been shown to impact clearance of the ADC leading to lower exposures and potential confounding of E-R analyses [13, 14, 16]. It is important to note that IgG levels in this analysis were based on total IgG levels and not specific to an IgG myeloma subtype, thereby limiting the clinical applicability of this finding. The association between Cycle 1 belantamab mafodotin efficacy endpoints and prior anti-CD38 treatment should also be interpreted with caution, as most patients (61 of 64 patients) who reported prior anti-CD38 treatment were in the DREAMM-6 Arm B study. Regarding Cycle 1 belantamab mafodotin safety endpoints, no covariates were associated with the probability of, or time to, grade ≥2 or grade ≥3 ocular AEs.

Strengths of this analysis include that it had a large patient population comprising pooled populations from two clinical studies (one a phase III study) with similar patient populations, study assessments, and overlap in timing; this allowed for incorporation of a wide range of dosing schedules/regimens, enabling a robust E-R analysis. Inclusion of multiple dose levels for belantamab mafodotin provided additional strength to the analysis, as utilization of a single dose level can lead to potential confounding of the E-R relationship [17]. This analysis examined Cycle 1-related belantamab mafodotin exposure metrics and their association with safety events and responses, most of which have been shown to occur within the first three cycles of treatment [5, 18, 19]. Clinical data suggests that belantamab mafodotin efficacy (including PFS) is preserved despite dose reductions and delays, mitigating the risk of subsequent doses impacting PFS and depth of responses [19]. The use of Cycle 1 exposure metrics mitigates confounding variables that can be caused by the time-dependent clearance, and reduces bias from dose modifications or exposure accumulation occurring after the first dose of belantamab mafodotin [14, 20], and is a further strength of the analysis. While other exposure metrics are available (such as time-to-event C_avg_), these were not selected as they have been shown to lead to biased estimates. C_avg_ over the first cycle remains the recommended exposure metric for E-R analyses particularly when the PK of a drug is associated with disease factors [21, 22]. In future analyses, more sophisticated modeling techniques that account for the entire time course of exposure and efficacy/safety assessments may lead to more robust identification of the underlying E-R relationship, though these would also require additional assumptions regarding the adequacy of the parametric models used for the description of the time-course.

While combining data from two separate studies may have limitations related to differences in patient populations and study designs, there were no clinically relevant differences in patient demographics and disease characteristics in the PK populations between the studies included in this analysis, with the exception of prior anti-CD38 treatment. The overall patient population was broadly representative of a population of patients with RRMM, though Black patients were under-represented, which is reported to be a common occurrence across trials in patients with MM [23, 24]. Data on anti-CD38 refractoriness in either study could be an important consideration; however, the number of patients expected to be anti-CD38 refractory was anticipated to be too small for evaluation in the E-R analyses.

Analysis of the BVd combination regimen meant it was not possible to identify the E-R relationship of belantamab mafodotin alone, as the analyses were confounded by the other therapies included in the regimen. The effect of the combination regimen may be further evaluated in the future, in a meta-analysis of the available E-R data, accounting for differences in disease status of the patients in each of the suite of DREAMM trials.

In conclusion, findings from the E-R analyses presented here support those of previous studies and indicate that higher belantamab mafodotin starting doses are associated with a higher probability of clinical response and a higher risk of OEFs, but not with the more clinically meaningful safety parameters of grade ≥2/ ≥3 ocular AEs (per CTCAE grade) or BCVA worsening to 20/50 or worse in both eyes. An initial belantamab mafodotin dose of 2.5 mg/kg, as used in the DREAMM-7 study, optimizes efficacy and tolerability compared with the 1.9 mg/kg dose, which was associated with reduced efficacy without a substantial reduction in AEs. Altogether, BVd represents a potential standard of care option with high efficacy [5], manageable safety, and a convenient Q3W dosing schedule for patients with RRMM in the second-line setting and beyond.

## Supplementary information

Supplemental material

## Data Availability

GSK makes available anonymized individual participant data and associated documents from interventional clinical studies that evaluate medicines, on approval of proposals submitted to: https://www.gsk-studyregister.com/en/. To access data for other types of GSK sponsored research, for study documents without patient-level data and for clinical studies not listed, please submit an enquiry via the website.
