# Supplementary material for: Exposure-response analyses for belantamab mafodotin in combination with bortezomib and dexamethasone in patients with relapsed/refractory multiple myeloma from DREAMM-6 Arm B and DREAMM-7
Source: Br J Cancer. 2026 Apr 13;135(1):60–8. doi: 10.1038/s41416-026-03437-7 (PMC13270149; doi:10.1038/s41416-026-03437-7)
Supplement: Supplementary file 1 — Supplemental material [file 41416_2026_3437_MOESM1_ESM.docx]

**Exposure-response analyses for belantamab mafodotin in combination with bortezomib and dexamethasone in patients with relapsed/refractory multiple myeloma from DREAMM-6 Arm B and DREAMM-7**

Theodoros Papathanasiou^1^, Xi Chen^2^, Fernando Carreno^2^ Astrid McKeown^3^, Sumita Roy-Ghanta^2^, Lydia Eccersley^4^, Ravi Kasinathan^5^, Nashita Patel^4^, Geraldine Ferron-Brady^2*^

**Supplementary Information**

**SUPPLEMENTARY METHODS**

**DREAMM-6 Arm B study**

*Study design*

DREAMM-6 Arm B is a phase I/II dose-escalation/expansion study. In the dose-escalation phase, eligible patients were assigned at the discretion of the investigator and received a single intravenous (IV) initial 2.5 mg/kg dose of belantamab mafodotin on Day 1 every 3 weeks (Q3W). Once safety and tolerability were confirmed, the next group of patients received an initial 3.4 mg/kg dose on Day 1 Q3W. In the dose-expansion phase, patients were assigned sequentially to 1 of 8 belantamab mafodotin cohorts; 1.9 mg/kg Q3W, 2.5 mg/kg Q3W, 3.4 mg/kg Q3W, 2.5 mg/kg split Q3W, 3.4 mg/kg split Q3W, 1.9 mg/kg Q6W, 2.5 mg/kg Q6W, and 2.5 mg/kg S/D to 1.9 mg/kg Q6W. For both phases, bortezomib 1.3 mg/m^2^ was administered by subcutaneous (SC) or IV infusion on Days 1, 4, 8, and 11, and dexamethasone 20 mg was administered orally or by IV infusion on Days 1, 2, 4, 5, 8, 9, 11, and 12 of each 21-day cycle, up to 8 cycles. Dose modifications (dose interruptions and reductions) for all study drugs were permitted after the completion of Cycle 1 to manage tolerability and adverse events (AEs), with the exception of reductions to the 1.9 mg/kg belantamab mafodotin doses in cohorts; 1.9 mg/kg Q3W, 1.9 mg/kg Q6W, and 2.5 mg/kg S/D to 1.9 mg/kg Q6W.

*Eligibility criteria*

| **Inclusion criteria** | **Exclusion criteria** |
| --- | --- |
| - ≥18 years - Confirmed diagnosis of MM per IMWG criteria - Measurable disease ECOG performance status of 0–2 - Received ≥1 prior LOT with documented disease progression during or after their most recent therapy - Received prior autologous SCT >100 days before study enrollment or were considered transplant ineligible - If female and of childbearing potential, were using a highly effective (failure rate <1%/year) contraceptive method | - Systemic anti-myeloma therapy (including systemic steroids) within ≤14 days, or plasmapheresis within 7 days prior to first dose of study drug - Use of an investigational drug within 14 days or five half-lives (whichever was longer) preceding first dose of study drug - Prior treatment with a monoclonal antibody within 30 days of receiving the first belantamab mafodotin dose - Prior allogenic SCT - Evidence of cardiovascular risk - Pregnant or lactating female - Current corneal disease except mild punctate keratopathy |

ECOG, Eastern Cooperative Oncology Group; IMWG, International Myeloma Working Group; LOT, line of therapy; MM, multiple myeloma; SCT, stem cell transplant.

*Endpoints*

| Primary endpoints | **Dose-escalation phase:** number and proportion of patients with a DLT and AEs (including SAEs)  **Dose-expansion phase:** AE, SAE, ORR (defined as the proportion of patients achieving a confirmed ≥PR according to IMWG response criteria) |
| --- | --- |
| Secondary endpoints | PK parameters during Cycle 1  Incidence of ADA against belantamab mafodotin  HRQoL  AESIs (including oAEs, ophthalmic examination findings, thrombocytopenia and IRR |
| Exploratory outcomes | ≥CR  ≥VGPR  TTR  TTBR  DOR  Time to progression  PFS  OS  Relationship between clinical response and sBCMA levels  MRD negativity for patients with ≥VGPR |

ADA, anti-drug antibodies; AE, adverse event; AESIs, AEs of special interest; ≥CR, complete response or better; DLT, dose-limiting toxicity, DOR, duration of response; HRQoL, health-related quality of life; IMWG, International Myeloma Working Group; IRR, infusion-related reactions; MRD, minimal residual disease; oAE, ocular adverse event; ORR, objective response rate; OS, overall survival; PFS, progression-free survival; ≥PR, partial response or better; SAE, serious adverse event; sBMCA, soluble B-cell maturation antigen; TTBR, time to best response; TTR, time to response; ≥VGPR, very good partial response or better

**Inclusion and exclusion criteria for DREAMM-7**

DREAMM-7 is a phase III study of BVd versus DVd in patients with a confirmed diagnosis of MM who have previously been treated with at least one line of therapy and have documented disease progression. Additionally, patients must have an Eastern Cooperative Oncology Group (ECOG) performance status of ≤2; ≥1 aspect of measurable disease, defined as one of the following: urine M-protein excretion ≥200 mg/24 hour, serum M-protein concentration ≥0.5g/dL or free light chain (FLC) level ≥10 mg/dL and an abnormal serum FLC ratio <0.26–<1.65; all prior treatment-related toxicities (defined by the National Cancer Institute Common Toxicity Criteria for Adverse Events v5.0) ≤grade 1 at time of enrollment, with the exception of alopecia; adequate organ function. Patients were excluded from the trial if they had disease that was refractory to anti-CD38 therapy or had prior exposure to BCMA therapy ^1^.

**Deltas for odds ratio and hazard ratio computation**

The following changes from the reference exposure and covariate values, i.e., deltas,

were used for the computation of odds ratios or hazard ratios:

- Cycle 1 belantamab mafodotin concentration at 21 days delta of 0.5 ug/mL, representing the difference between the predicted Cycle 1 belantamab mafodotin concentration at 21 days for a dose of 2.5 mg/kg and a dose of 1.9 mg/kg
- Cycle 1 belantamab mafodotin C_avg_ delta of 2.0 ug/mL, representing the approximate difference between the predicted Cycle 1 belantamab mafodotin C_avg_ for a dose of 2.5 mg/kg and a dose of 1.9 mg/kg
- Cycle 1 belantamab mafodotin C_max_ delta of 4.0 ug/mL, representing the approximate difference between the predicted belantamab mafodotin C_max_ for a dose of 2.5 mg/kg and a dose of 1.9 mg/kg
- Cycle 1 cys-mcMMAF C_max_ delta of 0.22 ng/mL, representing the difference between the predicted cys-mcMMAF C_max_ for a dose of 2.5 mg/kg and a dose of 1.9 mg/kg
- Cycle 1 cys-mcMMAF C_avg_ delta of 0.05 ng/mL, representing the difference between the predicted cys-mcMMAF C_avg_ for a dose of 2.5 mg/kg and a dose of 1.9 mg/kg
- Baseline IgG delta of 3 g/L, representing a 20% change from the typical value
- Baseline albumin delta of 8 g/L, representing a 20% change from the typical value
- Baseline sBCMA delta of 20 ng/mL, representing a 40% change from the typical value
- Baseline β2-microglobulin delta of 100 nmol/L, representing the approximate difference between quartiles
- Baseline LDH delta of 50 U/L, representing the approximate change from either the 1st quartile or 3rd quartile to the median
- Baseline platelet count delta of 25×10^9^/L, representing the difference between each grade of thrombocytopenia

**Statistical modeling, stepwise covariate selection, representative R implementation, and computational environment**

*Binary endpoints (logistic regression)*

Binary endpoints were analyzed using generalized linear models with a binomial distribution and logit link:

$$Y_{i}\sim\mathrm{Bernoulli}(p_{i}),\mathrm{logit}(p_{i})=\beta_{0}+\sum_{k} \beta_{k}X_{ik}.$$

Covariate selection was likelihood-based. Candidate covariates were first evaluated in univariate logistic regression models and compared to an intercept-only model using likelihood ratio tests (LRTs). For nested models $M_{R}$and $M_{F}$:

$$\Delta OFV=-2\{\mathcal{l}(\hat{\theta}_{R})-\mathcal{l}(\hat{\theta}_{F})\}\sim\chi_{\Delta df}^{2},$$

where $\mathcal{l}(\cdot)$is the maximized log-likelihood; $\Delta OFV$ corresponds to the change in deviance.

Forward selection was initiated from the null model by adding covariates meeting a prespecified entry criterion ($\alpha_{F}$). The resulting multivariable model was refined using backward elimination, sequentially removing covariates with LRT p-values exceeding the retention threshold ($\alpha_{B}$). Final model parameters are reported with 95% Confidence Intervals, and effect sizes are presented as odds ratios $\mathrm{OR}=\exp(\beta\Delta)$, with $\Delta=1$ unless otherwise stated.

*Representative R snippets for binary endpoints*

# Univariate screening

m0 <- glm(y ~ 1, data = dat, family = binomial())

m1 <- glm(y ~ cov1, data = dat, family = binomial())

anova(m0, m1, test = "Chisq")

# Backward step

dropterm(m_full, test = "Chisq")

# Odds ratio

OR <- exp(coef(m_final)["cov1"])

*Time-to-event endpoints (Cox proportional hazards)*

Time-to-event endpoints were analyzed using Cox proportional hazards models:

$$h(t\mid X_{i})=h_{0}(t)\exp\left( \sum_{k} \beta_{k}X_{ik} \right)$$

with parameters estimated by partial likelihood.

Covariate selection followed the same likelihood-based framework. Univariate Cox models were screened using LRTs based on partial log-likelihood. The objective function was defined as:

$$\mathrm{OFV}=-2\text{ }\mathcal{l}_{p}(\hat{\theta})$$

and covariate contribution was summarized by $\Delta OFV$(LRT statistic).

Forward selection added the covariate with the strongest improvement in fit ($p\leq\alpha_{F}$), followed by backward elimination removing covariates with $p>\alpha_{B}$. Final effects are reported as hazard ratios $\mathrm{HR}=\exp(\beta\Delta)$, with $\Delta=1$unless otherwise specified.

*Representative R snippets for time-to-event endpoints*

# Univariate screening

m1 <- coxph(Surv(time, event) ~ cov1, data = dat)

summary(m1)$logtest

# Forward / backward steps

addterm(m0, ~ cov1 + cov2, test = "Chisq")

dropterm(m_fwd, test = "Chisq")

# Hazard ratio

HR <- exp(coef(m_final)["cov1"])

*Computational environment*

R package versions that were used for the analysis were:

**R version:** 4.1.3 (2022-03-10)
**Platform:** x86_64-pc-linux-gnu (64-bit)
**Operating system:** Ubuntu 18.04.6 LTS

**Base packages**
stats, graphics, grDevices, utils, datasets, methods, base

**Attached packages**
table1 (1.4.2), survminer (0.4.9), survival (3.4-0), kableExtra (1.3.4), GGally (2.1.2),
psych (2.2.5), data.table (1.14.2), gridExtra (2.3), stringr (1.4.1), purrr (0.3.4),
knitr (1.40), broom (1.0.1), tidyr (1.2.0), dplyr (1.0.9), ggpubr (0.4.0),
ggplot2 (3.3.6), cowplot (1.1.1), magrittr (2.0.3), MASS (7.3-58.1),
nlme (3.1-159), mgcv (1.8-40), Matrix (1.4-1), car (3.1-0), Hmisc (4.7-1),
rmarkdown (2.16), rlang (1.0.5), tidyselect (1.1.2), lifecycle (1.0.1),
vctrs (0.4.1), tibble (3.1.8), zoo (1.8-10), jsonlite (1.8.0),
htmltools (0.5.3), scales (1.2.1), lattice (0.20-45)

**SUPPLEMENTARY FIGURES AND TABLES**

**Supplementary Table 1. Covariates assessed in the E-R analyses for DREAMM-6 Arm B and DREAMM-7**

| **Category** | **Covariates** |
| --- | --- |
| Demographics | Weight, body mass index, age, race*, gender, region |
| Baseline clinical status | Albumin, serum IgG, renal function category, hepatic function category by National Cancer Institute Index |
| Baseline disease status | Type of myeloma immunoglobulin (IgG versus others), cytogenetics risk, soluble BCMA, β_2_-microglobulin, lactate dehydrogenase, stage of disease, ECOG status, presence of extramedullary disease |
| Others | Number of prior lines of therapy, prior anti-CD38 treatment, prior bortezomib treatment, refractory to both immunomodulatory drugs and PI, planned dosing schedule |
| Specific to OEFs/ocular safety | Known history of dry eye per screening questionnaire, presence of keratopathy at baseline exam, baseline best-corrected visual acuity in better eye and in worse eye |
| Specific to thrombocytopenia | Baseline platelet counts |

*Race included evaluation of White, Asian, North-East Asian and Black/African American race.

BCMA, B-cell maturation antigen; ECOG, Eastern Cooperative Oncology Group; E-R, exposure-response; IgG, immunoglobulin G; OEF, ophthalmic exam findings; PI, proteasome inhibitor.

**Supplementary Table 2. Summary of results of efficacy exposure-response analyses for combined DREAMM-6 Arm B and DREAMM-7 analyses**

|  | **Univariate analysis** | | | **Final model** | |
| --- | --- | --- | --- | --- | --- |
| **Endpoint** | **Univariate covariates** | **ΔOFV** | **Univariate p-value** | **Parameter** | **HR/OR (95% CI)** |
| **Probability of overall response** | Extramedullary disease | 42.43 | <0.001 | Extramedullary disease | 0.0676 (0.0274, 0.154) |
|  | Belantamab mafodotin C_max_ | 31.09 | <0.001 | Belantamab mafodotin C_avg_ over 21 days post-dose | 1.97 (1.48, 2.69) |
|  | Baseline albumin | 23.20 | <0.001 |  |  |
|  | Belantamab mafodotin C_avg_ over 21 days post-dose | 22.37 | <0.001 |  |  |
|  | ISS stage I or stage II or stage III at screening | 25.59 | <0.001 |  |  |
|  | Baseline beta-2 microglobulin | 12.17 | <0.001 |  |  |
|  | Received prior therapy | 15.11 | 0.001 |  |  |
|  | Baseline LDH | 11.22 | 0.001 |  |  |
|  | Prior anti-CD38 treatment | 9.67 | 0.002 |  |  |
|  | EGOC PS ≥1 | 9.12 | 0.003 |  |  |
|  | Baseline sBCMA | 8.84 | 0.003 |  |  |
|  | Planned dosing schedule | 13.03 | 0.005 |  |  |
|  | ECOG PS 0 | 9.46 | 0.009 |  |  |
| **Probability of ≥VGPR** | Extramedullary disease | 31.31 | <0.001 | Extramedullary disease | 0.12  (0.0409,  0.298) |
|  | Belantamab mafodotin C_avg_ over 21 days post-dose | 27.28 | <0.001 | Belantamab mafodotin C_avg_ over 21 days post-dose | 1.95 (1.54, 2.51) |
|  | Prior anti-CD38 treatment | 22.39 | <0.001 | Prior anti-CD38 treatment | 0.23 (0.118, 0.436) |
|  | Received prior therapy | 21.56 | <0.001 |  |  |
|  | Baseline LDH | 15.94 | <0.001 |  |  |
|  | ≥1 prior therapy line | 14.01 | <0.001 |  |  |
|  | Baseline albumin | 12.29 | <0.001 |  |  |
|  | ISS stage I or stage II or stage III at screening | 14.79 | 0.001 |  |  |
|  | Baseline IgG | 7.45 | 0.006 |  |  |
|  | Cytogenetic risk | 6.92 | 0.009 |  |  |
| **Probability of ≥CR** | Prior anti-CD38 treatment | 25.43 | <0.001 | Prior anti-CD38 treatment | 0.102 (0.0301, 0.262) |
|  | Received prior therapy | 22.83 | <0.001 | Baseline IgG | 0.903 (0.853, 0.95) |
|  | ≥1 prior therapy line | 13.69 | <0.001 |  |  |
|  | Baseline IgG | 11.99 | 0.001 |  |  |
|  | Extramedullary disease | 10.04 | 0.002 |  |  |
|  | Baseline LDH | 8.95 | 0.003 |  |  |
|  | Baseline albumin | 7.57 | 0.006 |  |  |
| **PFS** | Prior anti-CD38 treatment | 39.18 | <0.001 | Prior anti-CD38 treatment | 2.82 (1.88, 4.22) |
|  | Received prior therapy | 37.00 | <0.001 | Extramedullary disease | 3.17 (2.06, 4.86) |
|  | Baseline LDH | 31.17 | <0.001 | Baseline LDH | 1.08 (1.05, 1.12) |
|  | Extramedullary disease | 29.89 | <0.001 |  |  |
|  | ISS stage I or stage II or stage III at screening | 28.99 | <0.001 |  |  |
|  | Planned dosing schedule | 27.71 | <0.001 |  |  |
|  | Baseline sBCMA | 13.84 | <0.001 |  |  |
|  | ≥1 prior therapy line | 11.70 | 0.001 |  |  |
|  | Baseline albumin | 11.19 | 0.001 |  |  |
|  | Baseline beta-2 microglobulin | 10.80 | 0.001 |  |  |
|  | Belantamab mafodotin C_max_ | 9.30 | 0.002 |  |  |
|  | Belantamab mafodotin C_avg_ over 21 days post-dose | 7.01 | 0.008 |  |  |
| **DOR** | Prior anti-CD38 treatment | 26.79 | <0.001 | Prior anti-CD38 treatment | 4.32 (2.64, 7.07) |
|  | Received prior therapy | 21.55 | <0.001 |  |  |
|  | Baseline LDH | 15.28 | <0.001 |  |  |
|  | Planned dosing schedule | 12.95 | 0.005 |  |  |
| **TTR** | Belantamab mafodotin C_tau_ at 21 days | 30.91 | <0.001 | C_avg_ over 21 days post-dose | 1.33 (1.19, 1.49) |
|  | Belantamab mafodotin C_avg_ over 21 days post-dose | 22.78 | <0.001 |  |  |
|  | Baseline IgG | 13.53 | <0.001 |  |  |
|  | IgG type multiple myeloma | 8.97 | 0.003 |  |  |

For the univariate analyses, the top 3 covariates resulting in ΔOFV >6.63 relative to the null model were included. Additionally, any covariates resulting in a ΔOFV >10.83 were included. Only the strongest exposure covariate was included; if Cycle 1 belantamab mafodotin Cavg was not the strongest exposure covariate, it was included for reference and comparison across the efficacy and safety endpoints.

C_avg_, average concentration; CI, confidence interval; C_max_, maximum concentration; CR, complete response; C_tau_, concentration at the end of a dosing interval; DOR, duration of response; ECOG PS, Eastern Cooperative Oncology Group performance status; HR, hazard ratio; IgG, immunoglobulin G; ISS, International Staging System; LDH, lactate dehydrogenase; PFS, progression-free survival; OFV, objective function value; OR, odds ratio; sBCMA, soluble baseline B-cell maturation antigen; TTR, time to response; ≥VGPR, very good partial response or better.

**Supplementary Table 3. Parameter estimates for combined DREAMM-6 Arm B and DREAMM-7 E-R analyses (final or alternative model)**

| **Model** | **Parameter** | **Units** | **Estimate** | **RSE (%)** | **95% CI** | **dOFV** | **Delta** | **HR/OR (95% CI)** |
| --- | --- | --- | --- | --- | --- | --- | --- | --- |
| **PFS** | Prior anti-CD38 treatment | — | 1.040 | 19.9 | (0.633, 1.44) | 21.88 | 1 | 2.82 (1.88, 4.22) |
|  | Extramedullary disease | — | 1.150 | 19.0 | (0.724, 1.58) | 21.96 | 1 | 3.17 (2.06, 4.86) |
|  | Baseline LDH | U/L | 0.002 | 21.0 | (0.00092, 0.00221) | 15.99 | 50 | 1.08 (1.05, 1.12) |
| **DOR** | Prior anti-CD38 treatment | — | 1.46 | 17.2 | (0.97, 1.96) | 26.79 | 1 | 4.32 (2.64, 7.07) |
| **Overall response*** | Intercept | — | -1.02 | 57.9 | (-2.21, 0.121) | N/A | N/A | — |
|  | Extramedullary disease | — | -2.69 | 16.2 | (-3.6, -1.87) | 43.85 | 1 | 0.0676^†^ (0.0274, 0.154) |
|  | C_avg_ over 21 days post-dose | μg/mL | 0.34 | 22.3 | (0.197, 0.494) | 23.78 | 2 | 1.97^†^ (1.48, 2.69) |
| **≥VGPR** | Intercept | — | -1.860 | 27.5 | (-2.89, -0.88) | N/A | N/A | — |
|  | Extramedullary disease | — | -2.120 | 23.5 | (-3.2, -1.21) | 23.63 | 1 | 0.12^†^ (0.0409, 0.298) |
|  | C_avg_ over 21 days post-dose | μg/mL | 0.333 | 18.7 | (0.215, 0.46) | 33.84 | 2 | 1.95^†^ (1.54, 2.51) |
|  | Prior anti-CD38 treatment | — | -1.470 | 22.6 | (-2.14, -0.829) | 20.81 | 1 | 0.23 (0.118, 0.436) |
| **≥CR** | Intercept | — | 0.007 | 2,802.6 | (-0.386, 0.404) | N/A | N/A | — |
|  | Prior anti-CD38 treatment | — | -2.280 | 23.6 | (-3.5, -1.34) | 30.26 | 1 | 0.102^†^ (0.0301, 0.262) |
|  | Baseline IgG | g/L | -0.034 | 26.7 | (-0.0528, -0.0171) | 16.82 | 3 | 0.903^†^ (0.853, 0.95) |
| **TTR*** | C_avg_ over 21 days post-dose | μg/mL | 0.142 | 20.4 | (0.0852, 0.199) | 22.78 | 2 | 1.33 (1.19, 1.49) |
| **MRD negativity (sCR/CR)^§^** | Null | — | — | — | — | — | — | — |

*Parameter estimates presented for alternative model which replaced the belantamab mafodotin C_max_ and concentration at 21 days (overall response and TTR, respectively) with C_avg_; ^†^odds ratio; ^§^analyses performed in DREAMM-7 alone.

C_avg_, average concentration; CI, confidence interval; C_max_, maximum plasma concentration; ≥CR, complete response or better; DOR, duration of response; E-R, exposure-response; IgG, immunoglobulin G; HR, hazard ratio; LDH, lactate dehydrogenase; N/A, not applicable; OFV, objective function value; probability of response; OR, odds ratio; PFS, progression-free survival; RSE, relative standard error; sCR, stringent complete response; TTR, time to response; ≥VGPR, very good partial response or better

**Supplementary Table 4. Summary of results of efficacy exposure-response analyses for the DREAMM-7 only analysis**

|  | **Univariate analysis** | | | **Final model** | |
| --- | --- | --- | --- | --- | --- |
| **Endpoint** | **Univariate covariates** | **ΔOFV** | **Univariate p-value** | **Parameter** | **HR/OR (95% CI)** |
| **Probability of overall response** | Extramedullary disease | 18.90 | <0.001 | Extramedullary disease | 0.0722 (0.0186, 0.235) |
|  | Belantamab mafodotin C_avg_ over 21 days post-dose | 9.67 | 0.002 |  |  |
| **Probability of ≥VGPR** | Belantamab mafodotin C_tau_ at 21 days | 20.44 | <0.001 | Belantamab mafodotin C_avg_ over 21 days post-dose | 1.77 (1.31, 2.44) |
|  | Belantamab mafodotin C_avg_ over 21 days post-dose | 17.33 | <0.001 | Extramedullary disease | 0.0992 (0.0149, 0.391) |
|  | Extramedullary disease | 15.16 | <0.001 |  |  |
|  | Baseline LDH | 8.68 | 0.003 |  |  |
|  | Cytogenetic risk | 8.68 | 0.003 |  |  |
| **Probability of ≥CR** | Baseline IgG | 15.09 | <0.001 | Baseline IgG | 0.896 (0.842, 0.949) |
|  | Belantamab mafodotin C_tau_ at 21 days | 9.17 | 0.002 |  |  |
|  | IgG type myeloma | 7.63 | 0.006 |  |  |
|  | Belantamab mafodotin C_avg_ over 21 days post-dose | 6.56 | 0.01 |  |  |
| **MRD negativity (sCR/CR)** | IgG type myeloma | 10.49 | 0.001 | Null model |  |
|  | Baseline IgG | 7.12 | 0.008 |  |  |
| **PFS** | Baseline LDH | 16.17 | <0.001 | Baseline LDH | 1.25 (1.14, 1.37) |
|  | Baseline sBCMA | 9.01 | 0.003 |  |  |
|  | ISS stage I or stage II or stage III at screening | 9.72 | 0.008 |  |  |
| **DOR** | Baseline LDH | 13.20 | <0.001 | Baseline LDH | 1.29 (1.15, 1.45) |
| **TTR** | Belantamab mafodotin C_tau_ at 21 days | 29.03 | <0.001 | Belantamab mafodotin C_avg_ over 21 days post-dose | 1.52 (1.29, 1.8) |
|  | Belantamab mafodotin C_avg_ over 21 days post-dose | 24.38 | <0.001 |  |  |
|  | IgG type myeloma | 10.13 | 0.001 |  |  |
|  | Baseline IgG | 9.09 | 0.003 |  |  |
|  | Cytogenetic risk | 6.79 | 0.009 |  |  |

For the univariate analyses, the top 3 covariates resulting in ΔOFV >6.63 relative to the null model were included. Additionally, any covariates resulting in a ΔOFV >10.83 were included. Only the strongest exposure covariate was included; if Cycle 1 belantamab mafodotin Cavg was not the strongest exposure covariate, it was included for reference and comparison across the efficacy and safety endpoints.

C_avg_, average concentration; CI, confidence interval; CR, complete response; C_tau_, concentration at the end of a dosing interval; DOR, duration of response; HR, hazard ratio; IgG, immunoglobulin G; ISS, International Staging System; LDH, lactate dehydrogenase; MRD, minimal residual disease; OFV, objective function value; OR, odds ratio; PFS, progression-free survival; sBCMA, soluble baseline B-cell maturation antigen; sCR, stringent complete response; TTR, time to response; ≥VGPR, very good partial response or better.

**Supplementary Table 5. Parameter estimates for final model or alternative final models for the DREAMM-7 E-R analyses**

| **Model** | **Parameter** | **Units** | **Estimate** | **RSE (%)** | **95% CI** | **dOFV** | **Delta** | **HR/OR (95% CI)** |
| --- | --- | --- | --- | --- | --- | --- | --- | --- |
| **PFS** | Baseline LDH | U/L | 0.004 | 20.9 | (0.00265, 0.00634) | 16.17 | 50 | 1.25 (1.14, 1.37) |
| **DOR** | Baseline LDH | U/L | 0.005 | 23.4 | (0.00276, 0.00744) | 13.2 | 50 | 1.29 (1.15, 1.45) |
| **Overall response** | Intercept | — | 1.82 | 10.5 | (1.46, 2.21) | N/A | N/A | — |
|  | Extramedullary disease | — | -2.63 | 24.0 | (-3.98, -1.45) | 18.9 | 1 | 0.0722^†^ (0.0186, 0.235) |
| **≥VGPR*** | Intercept | — | -1.500 | 42.7 | (-2.79, -0.266) | N/A | N/A | — |
|  | Extramedullary disease | — | -2.310 | 34.2 | (-4.2, -0.94) | 12.13 | 1 | 0.0992^†^ (0.0149, 0.391) |
|  | C_avg_ over 21 days  post-dose | μg/mL | 0.285 | 27.8 | (0.134, 0.446) | 14.31 | 2 | 1.77^†^ (1.31, 2.44) |
| **≥CR** | Intercept | — | 0.054 | 413.1 | (-0.383, 0.496) | N/A | N/A | — |
|  | Baseline IgG | g/L | -0.036 | 28.0 | (-0.0575, -0.0173) | 15.09 | 3 | 0.896^†^ (0.842, 0.949) |
| **TTR*** | C_avg_ over 21 days  post-dose | μg/mL | 0.211 | 20.1 | (0.128, 0.294) | 24.38 | 2 | 1.52 (1.29, 1.8) |
| **MRD negativity (sCR/CR)** | Null | — | — | — | — | — | — | — |

*Parameter estimates presented for alternative model which replaced belantamab mafodotin concentration at 21 days with C_avg_ (for ≥VGPR and TTR);
^†^odds ratio.

C_avg_, average concentration; CI, confidence interval; ≥CR, complete response or better; DOR, duration of response; E-R, exposure-response; HR, hazard ratio: IgG, immunoglobulin G; LDH, lactate dehydrogenase; MM, multiple myeloma; N/A, not applicable; OFV, objective function value; OR, odds ratio; PFS, progression-free survival; RSE, relative standard error; s, stringent; TTR, time to response; ≥VGPR, very good partial response or better.

**Supplementary Table 6. Frequency of safety endpoints**

| **Safety endpoint, n (%)** | **Overall  (N=349)** | **DREAMM-6 Arm B**  **(n=107)** | **DREAMM-7**  **(n=242)** |
| --- | --- | --- | --- |
| Dose modification due to AE | 314 (90.0) | 94 (87.9) | 220 (90.9) |
| Grade ≥3 TEAE | 312 (89.4) | 102 (95.3) | 210 (86.8) |
| Dose delay/interruption | 306 (87.7) | 99 (92.5) | 207 (85.5) |
| Grade ≥2 OEF (KVA) | 305 (87.4) | 96 (89.7) | 209 (86.4) |
| Grade ≥2 corneal exam finding (KVA) | 294 (84.2) | 96 (89.7) | 198 (81.8) |
| Grade ≥2 BCVA event (KVA) | 277 (79.4) | 83 (77.6) | 194 (80.2) |
| Grade ≥3 OEF (KVA) | 269 (77.1) | 82 (76.6) | 187 (77.3) |
| Dose reduction | 265 (75.9) | 86 (80.4) | 179 (74.0) |
| Grade ≥3 thrombocytopenia | 264 (75.9) | 86 (80.4) | 178 (73.9) |
| Grade ≥3 corneal exam finding (KVA) | 254 (72.8) | 79 (73.8) | 175 (72.3) |
| Grade ≥2 ocular AE (CTCAE) | 229 (65.6) | 87 (81.3) | 142 (58.7) |
| BCVA worsening in one eye (to 20/50 or worse) | 155 (64.0) | N/A | 155 (64.0) |
| Grade ≥3 BCVA event (KVA) | 196 (56.2) | 58 (54.2) | 138 (57.0) |
| BCVA worsening* | 166 (47.6) | 55 (51.4) | 111 (45.9) |
| BCVA worsening of both eyes (to 20/50 or worse) | 105 (43.4) | N/A | 105 (43.4) |
| Grade ≥3 ocular AE (CTCAE) | 140 (40.1) | 62 (57.9) | 78 (32.2) |
| Dose discontinuation | 70 (20.1) | 14 (13.1) | 56 (23.1) |
| IRR | 18 (5.2) | 14 (13.1) | 4 (1.7) |
| Fatal SAE | 11 (3.2) | 4 (3.7) | 7 (2.9) |

*A ≥0.3-point worsening in logMar (better eye).

AE, adverse event; BCVA, best-corrected visual acuity; CTCAE, Common Terminology Criteria for Adverse Events; IRR, infusion-related reaction; KVA, keratopathy and visual acuity; logMAR, logarithm of the minimum angle of resolution; N/A, not applicable/not collected; OEF, ophthalmic exam finding; SAE, serious adverse event; TEAE, treatment-emergent adverse event.

**Supplementary Table 7. Summary of safety E-R analyses**

|  |  | **Univariate analysis** | | | **Final model** | |
| --- | --- | --- | --- | --- | --- | --- |
| **Endpoint** | **Population** | **Univariate covariates** | **ΔOFV** | **Univariate p-value** | **Parameter** | **OR or HR  (95% CI)*** |
| Probability of grade ≥2 ocular AEs (CTCAE grade) | DREAMM-7 | NA | NA | NA | Null model |  |
| Time to first grade ≥2 ocular AE (CTCAE grade) | DREAMM-7 | NA | NA | NA | Null model |  |
| Probability of grade ≥3 ocular AEs (CTCAE grade) | DREAMM-7 | Presence of keratopathy at baseline | 8.26 | 0.004 | Null model |  |
| Time to first grade ≥3 ocular AE (CTCAE grade) | DREAMM-7 | Presence of keratopathy at baseline | 6.68 | 0.01 | Null model |  |
| Probability of grade ≥2 OEF (KVA scale) | DREAMM-6 Arm B and DREAMM-7 | Belantamab mafodotin C_avg_ | 36.42 | <0.001 | Belantamab mafodotin C_avg_ | 2.84 (1.96, 4.27) |
|  |  | Baseline albumin | 19.03 | <0.001 |  |  |
|  |  | Baseline sBCMA | 18.94 | <0.001 |  |  |
|  |  | Baseline LDH | 11.64 | 0.001 |  |  |
|  |  | ECOG PS 0 | 10.65 | 0.005 |  |  |
|  |  | ECOG PS ≥1 | 10.61 | 0.001 |  |  |
|  |  | ISS stage I or stage II or stage III at screening | 10.6 | 0.005 |  |  |
|  |  | Baseline beta-2 microglobulin | 8.17 | 0.004 |  |  |
| Time to first grade ≥2 OEF (KVA scale) | DREAMM-6 Arm B and DREAMM-7 | Belantamab mafodotin C_avg_ | 49.79 | <0.001 | Belantamab mafodotin C_avg_ | 1.49 (1.34, 1.66) |
|  |  | Baseline IgG | 15.28 | <0.001 |  |  |
|  |  | Baseline sBCMA | 12.65 | <0.001 |  |  |
|  |  | Baseline weight | 11.56 | 0.001 |  |  |
|  |  | Baseline best corrected visual acuity best eye | 10.04 | 0.002 |  |  |
|  |  | Prior anti-CD38 treatment | 8.91 | 0.003 |  |  |
|  |  | Baseline BMI | 7.39 | 0.007 |  |  |
|  |  | Residing in Europe | 6.8 | 0.009 |  |  |
| Probability of grade ≥3 OEF (KVA scale) | DREAMM-6 Arm B and DREAMM-7 | Belantamab mafodotin C_avg_ | 31.38 | <0.001 | Belantamab mafodotin C_avg_ | 2.05 (1.57, 2.72) |
|  |  | ISS stage I or stage II or stage III at screening | 18.73 | <0.001 |  |  |
|  |  | Baseline sBCMA | 16.45 | <0.001 |  |  |
|  |  | Baseline LDH | 13.17 | <0.001 |  |  |
|  |  | Baseline beta-2 microglobulin | 13.13 | <0.001 |  |  |
|  |  | ECOG PS 0 | 11.02 | 0.004 |  |  |
|  |  | Baseline albumin | 10.96 | 0.001 |  |  |
|  |  | ECOG PS ≥1 | 10.93 | 0.001 |  |  |
|  |  | Extramedullary disease | 9.95 | 0.002 |  |  |
| Time to first grade ≥3 OEF (KVA scale) | DREAMM-6 Arm B and DREAMM-7 | Belantamab mafodotin C_avg_ | 40.2 | <0.001 | Belantamab mafodotin C_avg_ | 1.48 (1.32, 1.67) |
|  |  | Residing in Europe | 12.58 | <0.001 |  |  |
|  |  | Baseline BMI | 9.29 | 0.002 |  |  |
|  |  | Baseline IgG | 9.16 | 0.002 |  |  |
|  |  | Baseline weight | 9.02 | 0.003 |  |  |
|  |  | Baseline sBCMA | 8.91 | 0.003 |  |  |
| Probability of grade ≥2 BCVA event (KVA scale) | DREAMM-6 Arm B and DREAMM-7 | Belantamab mafodotin C_max_ | 45.33 | <0.001 | Belantamab mafodotin C_avg_ | 2.49 (1.85, 3.44) |
|  |  | Belantamab mafodotin C_avg_ | 43.13 | <0.001 |  |  |
|  |  | Baseline albumin | 19.61 | <0.001 |  |  |
|  |  | Baseline sBCMA | 19.34 | <0.001 |  |  |
|  |  | ECOG PS 0 | 16.81 | <0.001 |  |  |
|  |  | Baseline beta-2 microglobulin | 16.18 | <0.001 |  |  |
|  |  | ECOG PS ≥1 | 15.93 | <0.001 |  |  |
|  |  | ISS stage I or stage II or stage III at screening | 15.81 | <0.001 |  |  |
|  |  | Baseline LDH | 12.87 | <0.001 |  |  |
|  |  | Planned dosing schedule | 11.79 | 0.008 |  |  |
|  |  | ISS stage I or stage II/III at screening | 8.81 | 0.003 |  |  |
|  |  | Extramedullary disease | 7.71 | 0.006 |  |  |
| Time to first grade ≥2 BCVA event (KVA scale) | DREAMM-6 Arm B and DREAMM-7 | Belantamab mafodotin C_avg_ | 35.22 | <0.001 | Belantamab mafodotin C_avg_ | 1.41 (1.26, 1.58) |
|  |  | Baseline albumin | 13.62 | <0.001 |  |  |
|  |  | Baseline BMI | 8.62 | 0.003 |  |  |
|  |  | Baseline IgG | 7.22 | 0.007 |  |  |
| Probability of grade ≥3 BCVA event (KVA scale) | DREAMM-6 Arm B and DREAMM-7 | Belantamab mafodotin C_avg_ | 19.52 | <0.001 | Belantamab mafodotin C_avg_ | 1.56 (1.27, 1.93) |
|  |  | Baseline sBCMA | 10.7 | 0.001 |  |  |
|  |  | Baseline albumin | 9.41 | 0.002 |  |  |
|  |  | Baseline IgG | 8.36 | 0.004 |  |  |
|  |  | Extramedullary disease | 7.54 | 0.006 |  |  |
| Time to first grade ≥3 BCVA event (KVA scale) | DREAMM-6 Arm B and DREAMM-7 | Planned dosing schedule | 28.74 | <0.001 | Split dosing schedule^†^ | 0.285 (0.138, 0.586) |
|  |  | Prior anti-CD38 treatment | 16.31 | <0.001 | Stretch dosing schedule^†^ | 0.574 (0.326, 1.01) |
|  |  | Belantamab mafodotin C_max_ | 10.04 | 0.002 | Stepdown stretch dosing schedule^†^ | 0.286 (0.117, 0.696) |
|  |  | Baseline albumin | 7.96 | 0.005 |  |  |
|  |  | Belantamab mafodotin C_avg_ | 0.88 | 0.349 |  |  |
| Probability of grade ≥2 corneal exam finding (KVA scale) | DREAMM-6 Arm B and DREAMM-7 | Belantamab mafodotin C_avg_ | 24 | <0.001 | Belantamab mafodotin C_avg_ | 2.06 (1.52, 2.86) |
|  |  | Baseline sBCMA | 12.6 | <0.001 |  |  |
|  |  | Baseline albumin | 11.34 | 0.001 |  |  |
|  |  | Baseline LDH | 9.69 | 0.002 |  |  |
| Time to first grade ≥2 corneal exam finding (KVA scale) | DREAMM-6 Arm B and DREAMM-7 | Belantamab mafodotin C_avg_ | 36.65 | <0.001 | Belantamab mafodotin C_avg_ | 1.45 (1.29, 1.62) |
|  |  | Prior anti-CD38 treatment | 19.48 | <0.001 | Split dosing schedule^†^ | 1.3 (0.807, 2.11) |
|  |  | Planned dosing schedule | 18.86 | <0.001 | Stepdown stretch dosing schedule^†^ | 2.55 (1.42, 4.59) |
|  |  | Baseline IgG | 13.86 | <0.001 | Stretch dosing schedule^†^ | 2.72 (1.7, 4.35) |
|  |  | Received prior therapy | 10.34 | 0.006 |  |  |
|  |  | IgG type multiple myeloma | 7.14 | 0.008 |  |  |
|  |  | Baseline weight | 7.03 | 0.008 |  |  |
| Probability of grade ≥3 corneal exam finding (KVA scale) | DREAMM-6 Arm B and DREAMM-7 | Belantamab mafodotin C_max_ | 26.58 | <0.001 | Belantamab mafodotin C_avg_ | 1.78 (1.4, 2.29) |
|  |  | Belantamab mafodotin C_avg_ | 24.29 | <0.001 |  |  |
|  |  | ISS stage I or stage II or stage III at screening | 17.35 | <0.001 |  |  |
|  |  | Baseline sBCMA | 16.45 | <0.001 |  |  |
|  |  | Baseline LDH | 15.79 | <0.001 |  |  |
|  |  | Baseline beta-2 microglobulin | 10.77 | 0.001 |  |  |
|  |  | Baseline albumin | 8.38 | 0.004 |  |  |
|  |  | Extramedullary disease | 8.1 | 0.004 |  |  |
|  |  | ECOG PS ≥1 | 7.53 | 0.006 |  |  |
| Time to first grade ≥3 corneal exam finding (KVA scale) | DREAMM-6 Arm B and DREAMM-7 | Belantamab mafodotin C_avg_ | 25.14 | <0.001 | Belantamab mafodotin C_avg_ | 1.34 (1.19, 1.52) |
|  |  | Residing in Europe | 16.04 | <0.001 | Residing in Europe | 0.624 (0.481, 0.809) |
|  |  | Baseline weight | 7.79 | 0.005 |  |  |
|  |  | IgG type multiple myeloma | 7.25 | 0.007 |  |  |
| Probability of worsening in best corrected visual acuity (BCVA) as defined by ∆logMar ≥0.3 in the better-seeing eye | DREAMM-6 Arm B and DREAMM-7 | Belantamab mafodotin C_avg_ | 19.27 | <0.001 | Belantamab mafodotin C_avg_ | 1.54 (1.27, 1.9) |
|  |  | Baseline albumin | 12.98 | <0.001 |  |  |
|  |  | Baseline beta-2 microglobulin | 8.63 | 0.003 |  |  |
|  |  | Baseline sBCMA | 8.32 | 0.004 |  |  |
| Probability of BCVA worsening in one eye to 20/50 or worse | DREAMM-7 | Baseline best corrected visual acuity worst eye | 13.11 | <0.001 | Baseline BCVA  (worst eye) | 2.49 (1.48, 4.65) |
|  |  | Cys-mcMMAF C_max_ | 11.94 | 0.001 | Cycle 1 cys-mcMMAF C_max_ | 0.785 (0.669, 0.903) |
|  |  | Baseline sBCMA | 7.69 | 0.006 |  |  |
|  |  | Presence of keratopathy at baseline | 7.18 | 0.007 |  |  |
|  |  | Belantamab mafodotin C_avg_ | 4.44 | 0.035 |  |  |
| Probability of worsening of BCVA in both eyes to 20/50 or worse | DREAMM-7 | Baseline best corrected visual acuity best eye | 18.82 | <0.001 | Baseline BCVA  (best eye) | 5.02 (2.21, 12.8) |
|  |  | Baseline best corrected visual acuity worst eye | 10.72 | 0.001 |  |  |
|  |  | Cys-mcMMAF C_avg_ | 7.44 | 0.006 |  |  |
|  |  | Belantamab mafodotin C_avg_ | 5.57 | 0.018 |  |  |
| Probability of occurrence of grade ≥3 thrombocytopenia | DREAMM-6 Arm B and DREAMM-7 | Baseline platelet count | 53.91 | <0.001 | Baseline platelet count | 0.684 (0.607, 0.763) |
|  |  | Asian race | 11.29 | 0.001 |  |  |
|  |  | Baseline age group | 9.77 | 0.008 |  |  |
|  |  | Residing in North East Asia | 8.13 | 0.004 |  |  |
| Probability of occurrence of grade 3–4 TEAEs | DREAMM-6 Arm B and DREAMM-7 | NA | NA | NA | Null model |  |
| Probability of dose discontinuation | DREAMM-6 Arm B and DREAMM-7 | ECOG PS 0 | 12.04 | 0.002 | Null model |  |
| Probability of dose reduction | DREAMM-6 Arm B and DREAMM-7 | ECOG PS ≥1 | 10.54 | 0.001 | Null model |  |
|  |  | History of dry eye | 8.23 | 0.004 |  |  |
|  |  | Baseline best corrected visual acuity best eye | 7.27 | 0.007 |  |  |
|  |  | Residing in Europe | 7.27 | 0.007 |  |  |
| Probability of dose delay/interruption | DREAMM-6 Arm B and DREAMM-7 | Belantamab mafodotin C_max_ | 13.43 | <0.001 | Belantamab mafodotin C_avg_ | 1.73 (1.26, 2.42) |
|  |  | ECOG PS 0 | 12.64 | 0.002 |  |  |
|  |  | Belantamab mafodotin C_avg_ | 12.04 | 0.001 |  |  |
|  |  | Baseline LDH | 11.53 | 0.001 |  |  |
|  |  | ECOG PS ≥1 | 9.75 | 0.002 |  |  |

For the univariate analyses, the top 3 covariates resulting in ΔOFV >6.63 relative to the null model were included. Additionally, any covariates resulting in a ΔOFV >10.83 were included. Only the strongest exposure covariate was included; if Cycle 1 belantamab mafodotin Cavg was not the strongest exposure covariate, it was included for reference and comparison across the efficacy and safety endpoints.

*OR or HR relate to specific delta for each parameter. ^†^Split: dosing split 50:50 between Days 1 and 8 Q3W. Stretch: Q6W. Stepdown stretch: Q6W with planned dose reduction at Cycle 3.

BCVA, best-corrected visual acuity; BMI, body mass index; C_avg_, average concentration; CI, confidence interval; C_max_, maximum plasma concentration; CTCAE, Common Terminology Criteria for Adverse Events; cys-mc, protease-resistant maleimidocaproyl linker; ECOG PS, Eastern Cooperative Oncology Group performance status; E-R, exposure-response; HR, hazard ratio; ISS, International Staging System; KVA, keratopathy and visual acuity; LDH, lactate dehydrogenase; logMAR, logarithm of the minimum angle of resolution; MMAF, monomethyl auristatin F; OEF, ophthalmic exam finding; OR, odds ratio; sBCMA, soluble B-cell maturation antigen; TEAE, treatment-emergent adverse event.

**Supplementary Figure 1. PFS (A), TTR (B), and DOR (C) Kaplan-Meier plots by belantamab mafodotin C_avg_ quartiles (DREAMM-7)**

**A**


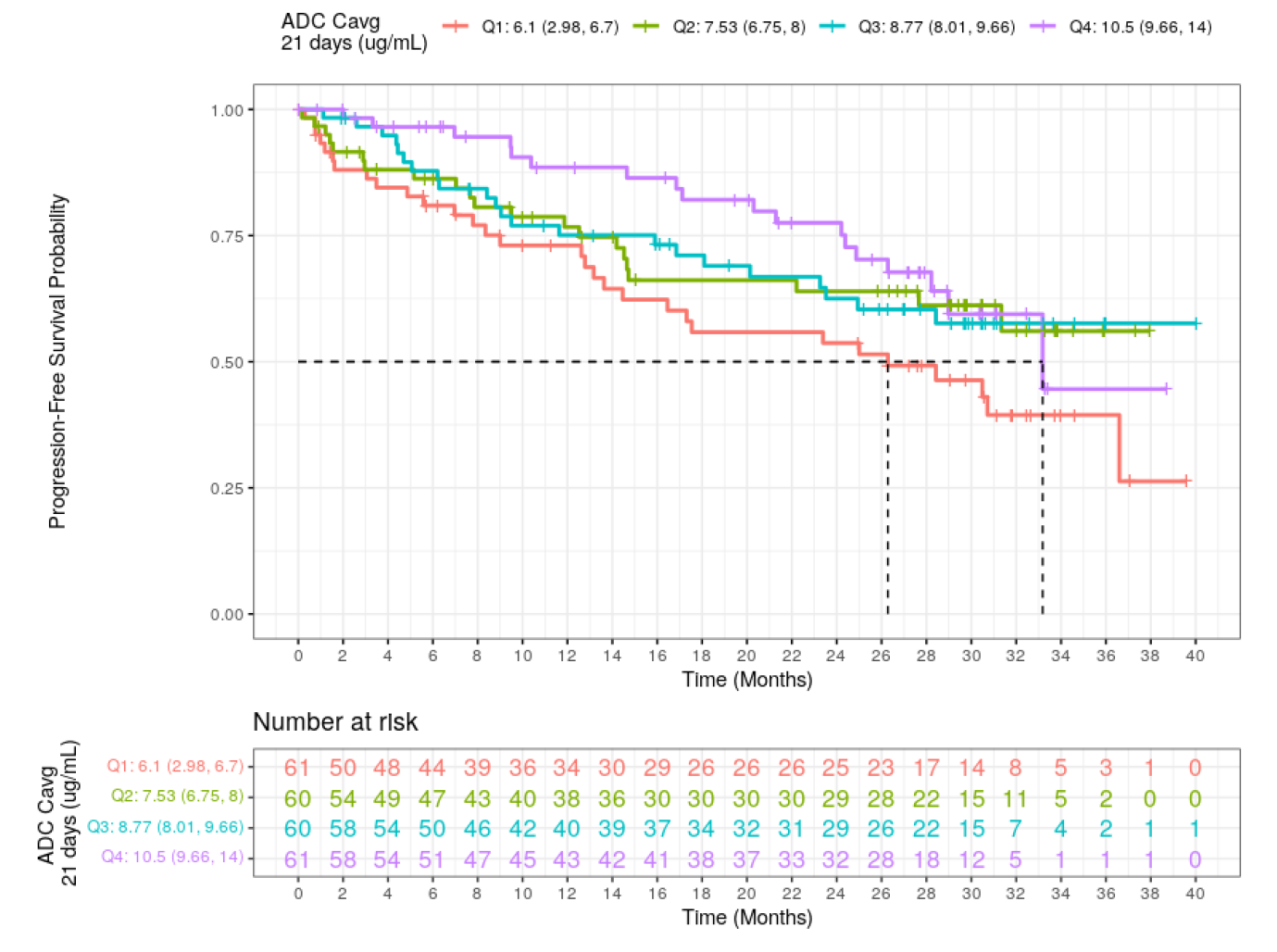


**B**


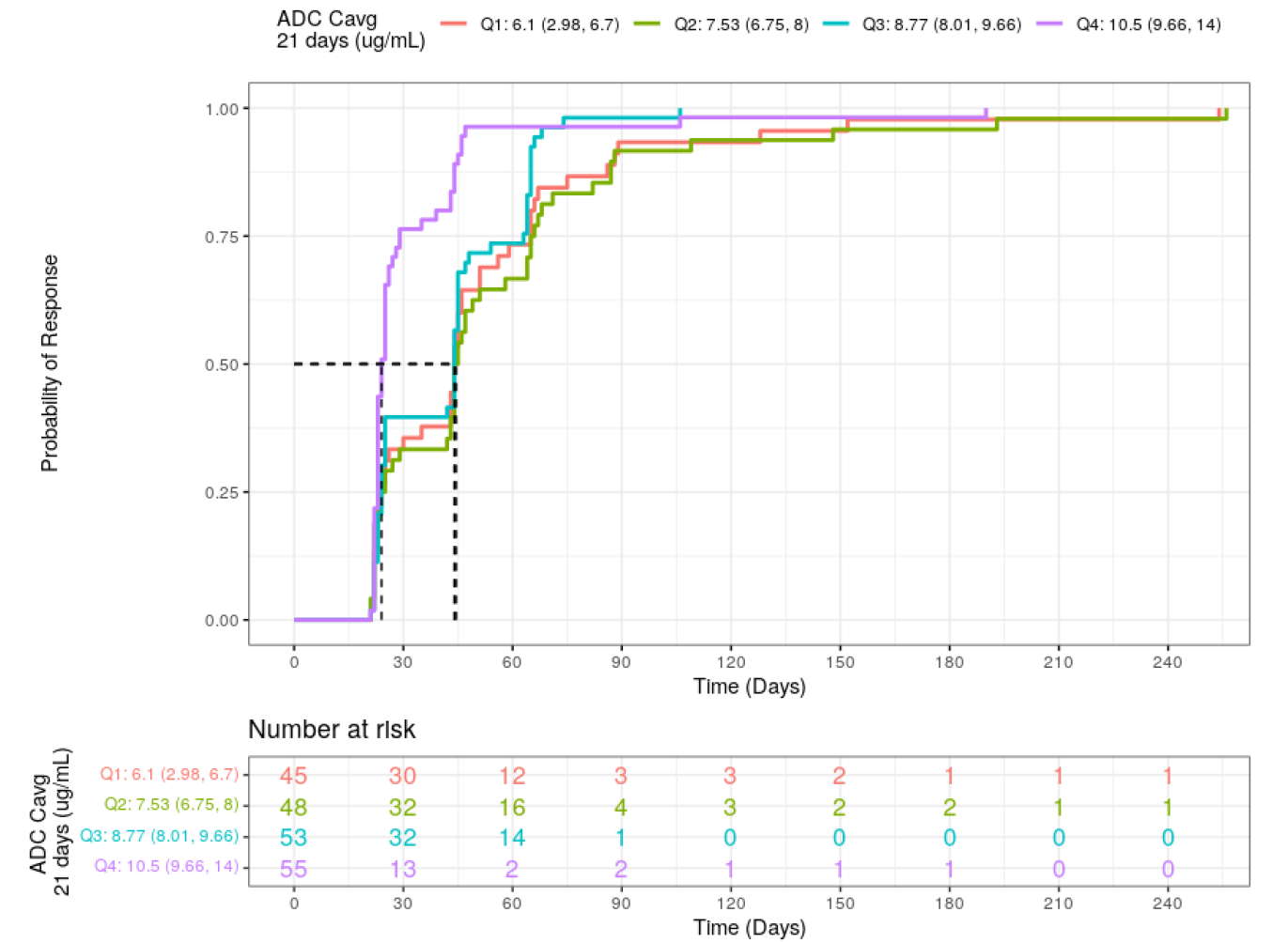


**C**


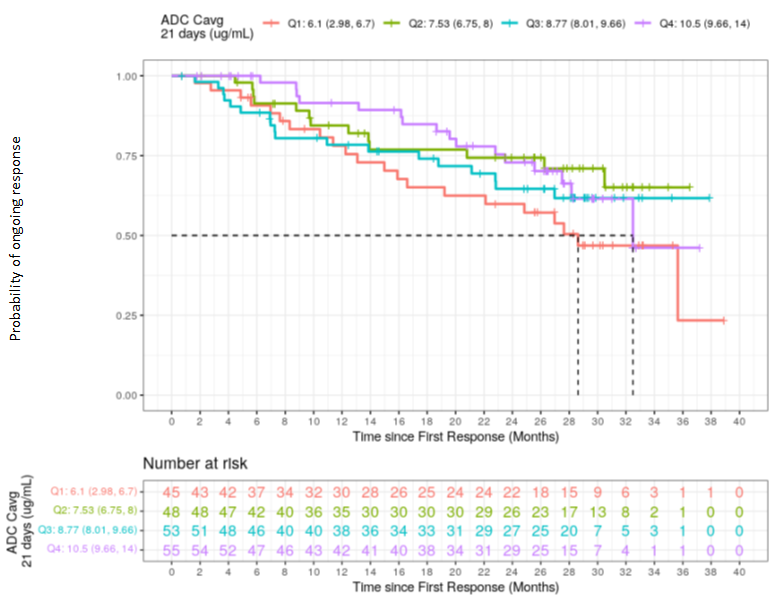


ADC, antibody-drug conjugate; C_avg_, average concentration; DOR, duration of response; PFS, progression-free survival; TTR, time to response; Q, quartile

**References**

**1.** Hungria V, Robak P, Hus M, et al. Belantamab Mafodotin, Bortezomib, and Dexamethasone for Multiple Myeloma. *N. Engl. J. Med.* 2024;391:393-407.
